# The Development Evaluation of Economic Zones in China

**DOI:** 10.3390/ijerph15010056

**Published:** 2018-01-02

**Authors:** Wei Liu, Hong-Bo Shi, Zhe Zhang, Sang-Bing Tsai, Yuming Zhai, Quan Chen, Jiangtao Wang

**Affiliations:** 1 Zhongshan Institute, University of Electronic Science and Technology of China, Zhongshan 528402, China; jiangtao-w@foxmail.com; 2 School of Economics and Management, Harbin Institute of Technology, Weihai 264209, China; dqytliu@gmail.com (W.L.); rand@sina.com (H.-B.S.); 3 Department of Urban Planning, University of Sydney, Sydney 2006, Australia; arch.zhangzhe@hotmail.com; 4 School of Economics and Management, Shanghai Institute of Technology, Shanghai 201418, China

**Keywords:** economic zones, MCDM model, DANP method, sustainable economic, sustainability

## Abstract

After the Chinese reform and opening up, the construction of economic zones, such as Special Economic Zones, Hi-tech Zones and Bonded Zones, has played an irreplaceable role in China’s economic development. Currently, against the background of Chinese economic transition, research on development evaluation of economic zones has become popular and necessary. Similar research usually focuses on one specific field, and the methods that are used to evaluate it are simple. This research aims to analyse the development evaluation of zones by synthesis. A new hybrid multiple criteria decision making (MCDM) model that combines the DEMATEL technique and the DANP method is proposed. After establishing the evaluation criterion system and acquiring data, the influential weights of dimensions and criteria can be calculated, which will be a guide for forming measures of development. Shandong Peninsula Blue Economic Zone is used in the empirical case analysis. The results show that Transportation Conditions, Industrial Structure and Business Climate are the main influencing criteria and measures based on these criteria are proposed.

## 1. Introduction

As the originator of the world’s economic zones, Leghoyn Freeport of Genoa Bay in Italy, which was founded in 1547, guided the development of the region’s economics. However, more and more countries (or regions) have had their own booming economic zones since “Silicon Valley” was founded by Stanford University in 1951. From this period, with the rapid development of science and technology, economic integration and marketization level improvement, the market competition is becoming more intense. Economic zones have received considerable attention [1]. There is no doubt that economic zones will play an irreplaceable role in economic development. 

Until now, the concept of economic zones has not been specific or standardized. The classification and interpretation of economic zones in different countries are not the same. German economist Zimmert summed up seven kinds of economic zones: the Bonded Zone, the Free Zone, the Foreign Trade Zone, the Export Processing Zone, the Special Economic Zone, the Enterprise Zone and the Bank-Free Zone [2]. In China, economic zones usually refer to a region associated with technological and economic development that accumulates a number of research institutes and industrial enterprises and forms a regional economic model of synchronous development of education, technology and economy [3]. Since its reform and opening up, China has built up a variety of economic zones, such as Special Economic Zones (SEZs), Hi-tech Zones, Economic and Technological Development Zones, Bonded Zones, etc. They have all contributed to the rapid development of China’s economy [4]. With the completion of the Blue Economic Zones in 2009 and the China (Shanghai) Pilot Free Trade Zone in 2013, China focused on the marine economy and comprehensive economic aggregates to guide economic development in the transition period. Furthermore, they have become the core power of economic development in the new period.

However, with the accelerating pace of economic globalization and the further deepening of China’s reform and opening up, China’s economic zones undertake an important responsibility and face huge pressures from external and internal directions [5,6]. Transformation and selecting the development direction of economic zones is also difficult. Therefore, it is particularly important to establish a scientific and effective evaluation method for economic zones. This will not only help the government accurately grasp the stage of economic zones and coordinate the national economy, but also to guide the prompt adjustment of development strategies for economic zones according to their status quo and utilize the actual characteristics of different economic zones in order to maximize development benefits [7,8].

This study aims to analyse the development evaluation of economic zones. A new hybrid MCDM Model that combines DEMATEL and DEMATEL-based ANP (DANP) is used in this research [9]. In this model, a scientific evaluation criterion system is established, first by representative dimensions and criteria, which influence the development of economic zones [10]. Then, the DEMATEL is used to build an influential network relations map (INRM). After that, the DEMATEL is combined with the Analytic Network Process (ANP) method to form DANP to obtain influential weights for each dimension and criterion in the evaluation structure [11,12]. Therefore, based on this method, it is helpful to handle the complex interactions and interdependencies among dimensions and criteria to make measures of development of economic zones.

The remainder of this paper is organized as follows: the next section reviews the literature on regional development evaluation and describes different evaluation methods. Section 3 illustrates the research methods. Section 4 provides an empirical case analysis that selects the Shandong Peninsula Blue Economic Zone for the case and discusses the results. Section 5 draws the conclusions.

## 2. Literature Review

### 2.1. Economic Zone System in China

An economic zone is the type of community system with a comprehensive development of economy, environment and technology. Economic zones not only have their own structure, but also reflect the developing functions in the continuing process of interactive influence with the external environment. Basically, the economic zone system is divided into three components: organization, operation and support. Organization refers to the management agency with decision-making, consulting, implementation and monitoring functions and business agency with developing, production and marketing functions. Operation refers to the mode and mechanism of various departments and enterprises. Support refers to the infrastructure construction and social integrated service system in the zones such as business, taxation, finance and insurance. The environment is an important factor for maintaining and ensuring the development of the system. The external environment of economic zones, referring to all the external factors and conditions that can have an effect and influence on the system, generally includes political, economic, cultural and ecological conditions from the regional, national and international levels. Thus, as a kind of economic system with specific structure, economic zone system reflects its economic, environmental and technological functions via input-output form, which transfer intelligence, money and other resources into the developing outcomes of economy, society and technology [5,6].

### 2.2. Literature Review

Many researchers have studied regional development evaluation in recent decades. Taken together, this research shows a dynamic development process. The methods of evaluation are different in different countries and regions in different stages of development [13,14,15].

Among the analytical methods, the two main aspects are qualitative analysis and quantitative analysis [16]. Qualitative analysis was often used in the early stages. The most classic research example is the work of Rodgers and Larson, which evaluated the development of Silicon Valley using a qualitative analysis of the history of development, venture capital, technical innovation, corporate networks and aspects of lifestyle to reveal the “agglomeration economies” condition of the formation of Silicon Valley [17]. Although this method of qualitative analysis is very difficult for development zones to make a comprehensive, scientific and objective evaluation, it has been made a milestone by creating the precedent for comprehensive evaluation.

After turning to quantitative methods, the scientific soundness and accuracy of research improved significantly. The research was reflected by building evaluation index systems to evaluate economic zones. In some outstanding economic zones, governments set up official evaluation index systems for reference. In 1995, Joint Venture Silicon Valley first published the “Index of Silicon Valley” as a comprehensive regional development evaluation report. Five main indexes, including talent, economy, society, environment and government, were used as the dimensions [18,19]. The Ministry of Science and Technology of China set up the indexes of China’s science and technology zones in three times. Technological innovation ability, economic development and innovation and entrepreneurship environment were chosen as the first level indexes.

On the selection of indexes and structure of evaluation index system, many researchers selected different kinds of indexes for evaluation. Markusen made an evaluation indicator system with three aspects (economics, industry, and region) and 14 criteria [20]. Eng studied the impact of the development of Cambridge Park on British economics through the diffusion of knowledge as an evaluation factor [21]. However, it lacks the aspect of development evaluation itself in theory. Hung selected 367 Taiwan’s high-tech enterprises as the research object and evaluated small and medium enterprises’ development by choosing the aspects of enterprise performance and enterprise profitability as the main indexes [22]. Chen considered the whole system of China’s Hi-tech zones and selected the evaluation indexes from three aspects: economy, environment, science and technology [23]. These evaluation indexes reflect the main ideas and characteristics of China’s economic zones at that time. Xie evaluated the development of high-tech industrial development zones in the Yangtze River Delta with 29 indexes from the economic ability, operational performance, science and technology innovation ability, opening ability to the outside and ecological efficiency [24]. However, the selection of indexes has great limitations [25].

On the evaluation methods, some classical methods such as Principal Component Analysis (PCA), Analytic Hierarchy Process (AHP), the factor analysis method, and the measurement score method were used frequently. Kitchen used PCA to evaluate Saskatoon’s social development in Canada with 38 indexes from aspects such as population, education, labour, income, etc. [26]. Li selected nine economic indexes and used PCA to evaluate regional economic development level of 15 vice provincial cities [27]. However, the number of indexes that used the PCA method is small, and the coverage is not wide. The conclusion is simply a description of the level of economic development and cannot evaluate the level of regional development well. Li determined the framework of development evaluation system of Hi-tech Zones and used the measurement score method to evaluate, but it does not analyse historical data [28]. Tian proposed the factor analysis method to evaluate Hi-tech Zones with five factors [29].

### 2.3. Evaluation Index System

By reviewing the previous literature, it is seen that current researchers have conducted extensive research on the development evaluation at different levels, from different perspectives and with different methods. They all achieve a certain effect, but there are still some problems to be solved and some aspects to be improved. For indexes, compared with the above indexes that are mostly statistical and derived from statistical data, the indexes used in this research are non-statistical indexes because the data in this research comes from the consciousness of experts. Its advantage is that non-statistical indexes can better analyse future development trends in order to make a forward-looking guidance of development at present. For evaluation methods, the lack of strong scientific methods, low effectiveness of data analysis and the absence of generally accepted and unified theory are still problems that need to be solved. A new method for regional evaluation needs to be developed.

As a result of literature review, three dimensions, including region (D_1_), economy (D_2_) and society (D_3_), are compiled. Each dimension includes lower-level criteria that originate from related indexes in previous literature and innovate in the actual situation of economic zones in China. The evaluation index system of economic zones which consists of three dimensions and 10 criteria is arranged in Table 1.

## 3. Methodology

### 3.1. Constructing a New Hybrid MCDM Model for Development Evaluation

**Figure 1:**
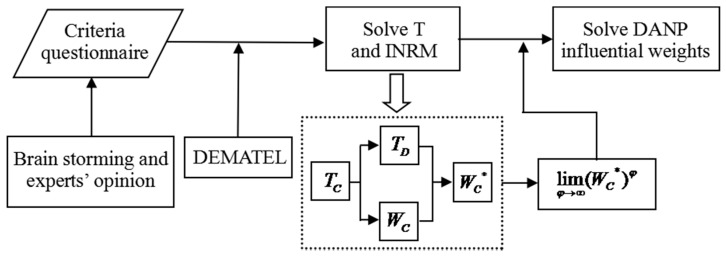
The research process of the new hybrid MCDM model.

This research uses the DEMATEL technique and combines a DANP method to establish a new hybrid MCDM Model to address the problems of interdependence and feedback among certain criteria. The DEMATEL technique is used to build an INRM, and the DANP is expected to obtain the influential weights using the basic concept of ANP [30,31,32,33,34,35]. The research processes are concisely illustrated in Figure 1.

### 3.2. The DEMATEL Technique for Building INRM

#### 3.2.1. Step 1: Calculate the Direct Influence Matrix by Scores

Knowledge-based experts were asked to assess the relationship between each mutual influence criterion in a personal sense using an integer scale ranging from 4 to 0, with the scores represented by natural language: “top influence (4)”, “high influence (3)”, “medium influence (2)”, “low influence (1)” and “absolutely no influence (0)”. The direct influence is indicated by pair-wise comparison. If the experts believe that criterion *i* has an effect and influence on criterion *j*, they should indicate this by *d_ij_*. Then, the matrix *D* = [*d_ij_*]_*n*×*n*_ of direct relationships can be represented as in Equation (1) [36,37,38,39,40]:

(1) $$D=\left(\begin{array}{ccccc} d_{11} & \cdots & d_{1j} & \cdots & d_{1n}\\ \vdots & & \vdots & & \vdots \\ d_{i1} & \cdots & d_{ij} & \cdots & d_{in}\\ \vdots & & \vdots & & \vdots \\ d_{n1} & \cdots & d_{nj} & \cdots & d_{nn} \end{array}\right)$$

#### 3.2.2. Step 2: Normalize the Direct Influence Matrix R

Using matrix D, the normalized matrix *R* = [*r_ij_*]_*n*×*n*_ can be calculated by Equations (2) and (3) as below:

(2) R=υ×D

(3) $$v=\min\left\{\frac{1}{\underset{i}{\max}\sum_{j=1}^{n}d_{ij}},\frac{1}{\underset{j}{\max}\sum_{i=1}^{n}d_{ij}}\right\},i,j\in \left\{1,2,\ldots ,n\right\}$$

#### 3.2.3. Step 3: Obtain the Total Influence Matrix T

The total influence matrix T for building influential network relationship map (INRM) can be obtained from Equation (4), in which *I* denotes the identity matrix [41,42,43,44,45]:

(4) $$T=R+R^{2}+\ldots +R^{X}=R(I-R^{X}){\left(I-R\right)}^{-1}$$

Then, $T=R{\left(I-R\right)}^{-1}$ when $\underset{X\to \infty }{\lim}R^{X}$, *T* = [0]_*n*×*n*_, where *R* = [*r_ij_*]_*n*×*n*_, 0 ≤ *R* ≤1, $0\leq \underset{i}{\sum }R_{ij}\leq 1$, $0\leq \underset{j}{\sum }R_{ij}\leq 1$, and at least one row or one column of the summation, but not each, equals one. Accordingly, $\underset{X\to \infty }{\lim}R^{X}={\left[0\right]}_{n\times n}$ is guaranteed naturally.

#### 3.2.4. Step 4: Analyse the Results and Build the INRM

After obtaining the total influence matrix T, its sum of rows and columns can be expressed respectively as vector *r* and vector *c*, which come from Equations (5) and (6):

(5) $$r=\left[\sum_{j=1}^{n}t_{ij}\right]={\left[t_{i}\right]}_{n\times 1}=(r_{1},\ldots ,r_{i},\ldots ,r_{n}{)}^{\prime },j\in \left\{1,2,\ldots ,n\right\}$$

(6) $$c={\left[\sum_{i=1}^{n}t_{ij}\right]}_{1\times n}{}^{\prime }={\left[t_{j}\right]}_{n\times 1}=(r_{1},\ldots ,r_{i},\ldots ,r_{n}{)}^{\prime },j\in \left\{1,2,\ldots ,n\right\}$$

In equations above the superscript’ denotes the transpose. Because of the same number of elements in vector *r* and vector *c*, *r* + *c* and *r* − *c* can establish two column vectors as Equations (7) and (8) [46,47,48,49]:

(7) $$r+c=(r_{1}+c_{1},\ldots ,r_{i}+c_{i},\ldots ,r_{n}+c_{n}{)}^{\prime },i\in \left\{1,2,\ldots ,n\right\}$$

(8) $$r-c=(r_{1}-c_{1},\ldots ,r_{i}-c_{i},\ldots ,r_{n}-c_{n}{)}^{\prime },i\in \left\{1,2,\ldots ,n\right\}$$

*r_i_* indicates the sum of the direct and indirect effects of criterion *i* on the other criteria. *c_j_* indicates the sum of the direct and indirect effects that criterion *j* has received from the other criteria. In addition, (*r_i_* + *c_j_*) indicates the degree of the total influences criterion *i* has in this system. Thus, if (*r_i_* − *c_j_*) is positive, then criterion *i* has a net influence on the other criteria. If (*r_i_* − *c_j_*) is negative, then criterion *i* is influenced by the other criteria in general. Therefore, a causal graph can be achieved by mapping the data set of (*r_i_* + *c_j_*, *r_i_* − *c_j_*), which is the so-called INRM. The INRM can provide a valuable idea for improvement on criterion.

### 3.3. Combing the ANP Method for Determining the Criterion’s Influence Weights

#### 3.3.1. Step 1: Find the Normalized Matrix $T_{C}^{a}$ Using the Dimensions and Take the Normalization of $T_{C}^{a11}$, for Instance

In this part, we specify the total influence matrix *T_C_* by the criteria and *T_D_* by the dimensions. $T_{C}={\left[t_{C}^{ij}\right]}_{n\times n}$, $T_{D}={\left[t_{D}^{ij}\right]}_{m\times m}$ and *T_D_* can be obtained from *T_C_*. After obtaining *T_C_*, the normalized total influence matrix $T_{C}^{a}$ for the criteria can be shown as Equation (9) [43,44,45,46]:

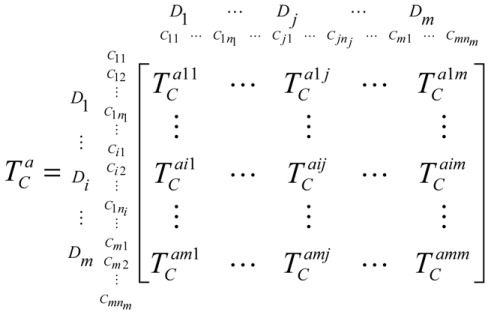
(9)


#### 3.3.2. Step 2: Obtain the Unweighted Supermatrix *W_C_*

The unweighted supermatrix *W_C_* can be obtained based on transposing the normalized total influence matrix $T_{C}^{a}$ using dimensions because of the interdependence between the relationships of the clusters and dimensions. Based on the basic concept of ANP, the supermatrix $W_{C}={\left(T_{C}^{a}\right)}^{\prime }$ is shown as Equation (10):
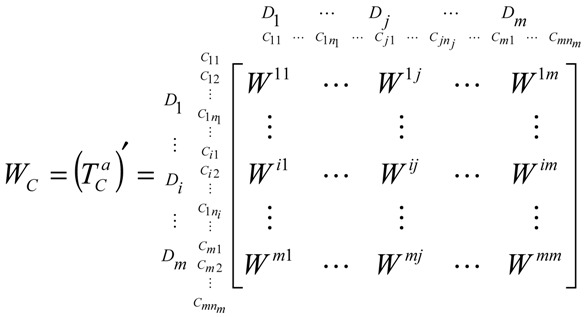
(10)

In this equation, the supermatrix *W_C_* has a case that its dimensions and criteria are independent if a blank or zero appears in the matrix.

#### 3.3.3. Step 3: Obtain the Weighted Normalized Supermatrix $W_{C}^{*}$

To obtain the weighted normalized supermatrix $W_{C}^{*}$, the unweighted supermatrix *W_C_* is the basis and the normalized total influence matrix $T_{D}^{a}$ is of the essence. We can get $W_{C}^{*}$ by multiplying $T_{D}^{a}$ by the unweighted supermatrix *W_C_*. Meanwhile, $T_{D}^{a}$ can be obtained by the total influence matrix *T_D_*. After normalizing the total influence matrix *T_D_*, we can obtain a new normalized total influence matrix $T_{D}^{a}$, which can be shown as Equation (11):

(11) $$\begin{array}{ll} T_{D}^{a} & =\left[\begin{array}{ccccc} \frac{t_{D}^{11}}{d_{1}} & \cdots & \frac{t_{D}^{1j}}{d_{1}} & \cdots & \frac{t_{D}^{1n}}{d_{1}}\\ \vdots & & \vdots & & \vdots \\ \frac{t_{D}^{i1}}{d_{i}} & \cdots & \frac{t_{D}^{ij}}{d_{i}} & \cdots & \frac{t_{D}^{in}}{d_{i}}\\ \vdots & & \vdots & & \vdots \\ \frac{t_{D}^{n1}}{d_{n}} & \cdots & \frac{t_{D}^{nj}}{d_{n}} & \cdots & \frac{t_{D}^{nn}}{d_{n}} \end{array}\right]\\ & =\left[\begin{array}{ccccc} t_{C}^{a11} & \cdots & t_{C}^{a1j} & \cdots & t_{C}^{a1n}\\ \vdots & & \vdots & & \vdots \\ t_{C}^{ai1} & \cdots & t_{C}^{aij} & \cdots & t_{C}^{ain}\\ \vdots & & \vdots & & \vdots \\ t_{C}^{an1} & \cdots & t_{C}^{anj} & \cdots & t_{C}^{ann} \end{array}\right] \end{array}$$

After obtaining the normalized total influence matrix $T_{D}^{a}$, we can calculate and obtain the new weighted supermatrix $W_{C}^{*}$ by the unweighted supermatrix *W_C_* . The result is shown in Equation (12):

(12) $$\begin{array}{ll} W_{C}^{*} & =T_{D}^{a}\times W\\ & =\left[\begin{array}{ccccc} t_{D}^{a11}\times W^{11} & \cdots & t_{D}^{a1j}\times W^{i1} & \cdots & t_{D}^{a1n}\times W^{n1}\\ t_{D}^{ai1}\times W^{1j} & \cdots & t_{D}^{aij}\times W^{ij} & \cdots & t_{D}^{a1n}\times W^{nj}\\ t_{D}^{an1}\times W^{1n} & \cdots & t_{D}^{anj}\times W^{in} & \cdots & t_{D}^{ann}\times W^{nn} \end{array}\right] \end{array}$$

#### 3.3.4. Step 4: Obtain the DANP

Limit the weighted supermatrix by raising it to a sufficiently large power φ until it converges and becomes a long-term stable supermatrix to obtain global priority vector, i.e., $\underset{\varphi \to \infty }{\lim}{\left(W_{C}^{*}\right)}^{\varphi }$, where φ represents any number of powers when φ→∞ and the influential weights *W* = (*W*_1_,…,*W_j_*,…,*W_n_*). Then, we can call these the DANP influential weights.

## 4. Empirical Study

The methodology established in this study aims to evaluate the development of economic zones in China. As the characteristics of each economic zone are heterogeneous and the weight of each criteria within evaluation system is different, we chose Shandong Peninsula Blue Economic Zone as the case to test the methodology. In this study, we use the software Matlab to establish and calculate the models directly [50,51,52].

### 4.1. Background of Research Object

The blue economic zone is a new concept of an economic zone in China. As a new type of coastal economic zone with marine characteristics, the blue economic zone aims to enhance the comprehensive economic strength and international competitiveness using the marine economy as the remarkable feature, marine resources as the fundamental way and modern marine industry as the leading force [53,54,55]. The first blue economic zone in China is Shandong Peninsula Blue Economic Zone, which was planned in 2009 and built in 2011. As the first example of a blue economic zone, Shandong Peninsula Blue Economic Zone has the conditions and advantages on infrastructure, investment environment, factor resource and economic foundation [56,57]. However, some problems have been gradually reflected during the process of development. On one hand, the advantages of marine natural resources and marine science and technology resources are obvious in Shandong Peninsula Blue Economic Zone, while in the actual development, competitive industries do not reach the maximum utilization and are in a lower proportion of the industrial structure. Therefore, the economic development efficiency of the whole zone has not improved significantly. On the other hand, the government has formulated many development strategies and policies in the process of construction. However, it lacks unified development ideas and effective policy orientation. This problem has led to fuzzy development strategy and policy guidance. The development potential of the Shandong Peninsula Blue Economic Zone is not completely liberalized, so a method for evaluation of its development is urgently needed. Therefore, it is very meaningful and useful to regard Shandong Peninsula Blue Economic Zone of Shandong Peninsula as a research objective in order to obtain development decisions from development evaluation.

### 4.2. Data Collection

The data in this research was collected from an expert forecasting questionnaire. Ten representative experts were selected to complete the questionnaire. To reflect the comprehensiveness and representativeness, the selected experts have the following characteristics: four university professors that have studied in this area for more than 10 years, three entrepreneurs that have set up the companies in this zone for more than three years, and three civil servants that have worked for government of this zone for more than three years. After collecting the 10 expert forecasting questionnaires, the data needed to be processed and integrated. Then, the average impact score of each criterion can be obtained. The initial influence matrix D is shown by Table 2.

### 4.3. Model Calculation Process

Using the initial influence matrix D, the normalized matrix R can be calculated and is shown in Table 3.

The total influence matrices *T_C_* of the criteria and *T_D_* of the dimensions are calculated and shown in Table 4 and Table 5, respectively.

Then, *r*, *c*, *r* + *c* and *r* − *c* are used for building the INRM are calculated and shown in Table 6.

The influence weights for the 10 criteria can be calculated using DANP, as shown in Table 7, Table 8 and Table 9.

## 5. Results and Discussions

Through the stable matrix of DANP $\underset{\varphi \to \infty }{\lim}{\left(W_{C}^{*}\right)}^{\varphi }$, the final results are sorted by Table 10.

According to the Table 10, it is easily seen that Transportation Conditions, Industrial Structure and Business Climate are the main influencing criteria, with results of 0.410, 0.392 and 0.392, respectively. Development Potential, Policy Support and Economic Scale are the less important criteria, with results of 0.344, 0.343 and 0.314, respectively. There are several important results in this research.

First, the result for Transportation Conditions is highest of all. It means that transportation conditions will be the most important index for improving the development of the Shandong Peninsula Blue Economic Zone. Therefore, the first suggestion is to improve the transportation conditions and strengthen the construction of the transportation system. As a region that has a long coastline, marine transportation is a unique advantage for the transportation system, so it is very important to construct large tonnage ports and improve port operational efficiency. To improve the ability of marine transportation, it is necessary to build up the fast convergence of marine transportation and other transportation modes, especially the railway transportation, and finally build marine transportation as the core of an integrated transportation system.

Second, as the second highest result, Industrial Structure is very valuable for the development of the Shandong Peninsula Blue Economic Zone. It is important to promote industrial development and accelerate the optimization of industrial structure. The zone’s development will not be possible without industrial development. On the one hand, industrial development patterns and industrial clusters need to be planned comprehensively and scientifically. Zones will select superior industries according to the actual situation in order to establish relevant supporting facilities and construct an integrated industrial chain.

Third, as far as the rest of criteria, the result of Business Climate is the highest. The Business Climate has two aspects: one is enterprise policy and the other is open environment. For enterprise policy, one must formulate some policies to attract the investment and construction of enterprises through the policy guidance, such as tax policies, to reduce the cost of business operations. For open environment, improving the openness of zones is important for the Business Climate. The Shandong Peninsula Blue Economic Zone has the geographical advantages of neighbouring South Korea and Japan and has a prominent historical and cultural environment for foreign trade. Therefore, the zone should take the initiative to attract foreign investment, play geopolitical advantages and promote Sino foreign cooperation. 

## 6. Conclusions

This research reflects precious values. First, the entirety of the research is embodied by a quantitative method. It inherits many advantages of existing studies in this area, such as the thoughts of criteria determination and system construction. On this basis, it improves the method and obtains better results. Second, the model uses the DEMATEL technique and combines it with a DANP method to establish a new hybrid MCDM model. In the above sections, it can be seen that the model has a rigorous mathematical logic that obtains convincing results. It is a good solution to solve the problem, which lacks the precision of the results by the research data. This is due to the first application, which combines the DEMATEL technique and the DANP method in the area of regional development evaluation. Third, as the research objective, the significance of the development of economic zones is self-evident. This research gives a method to identify the valuable dimensions and criteria and is a guide for managers to develop economic zones more effectively. Furthermore, this research has huge potential. As a method of regional development evaluation, this method can be used on economic zones. It can expand the research objective, and it will be applied to the development evaluation of all specific regions. Finally, this method hopefully will become a representative solution for solving such problems. 

There are several limitations to this research that require further study. First, the number of selected experts that are used for data collection from expert forecasting questionnaire needs to be expanded. More data samples can improve the credibility of research. Second, the selected experts should be more representative. The expert quality has a strong positive correlation with the results of the research. Finally, in the process of selecting dimensions and criteria, future researchers need to use a scientific method to establish a more practical evaluation index system that can improve the accuracy of the results.

## Figures and Tables

**Table 1:** The economic zones evaluation index system.

Dimensions	Criteria	Descriptions
**Region** (**D_1_**)	Transportation Conditions (C_11_)	Information about various transportation in one region.
	Resource Reserves (C_12_)	Natural resources in one region which matched with the regional industry.
	Ecological Protection (C_13_)	The present situation of the environment and protection measures.
**Economy** (**D_2_**)	Industrial Structure (C_21_)	The composition of each industry and the proportion relationship among industries.
	Economic Scale (C_22_)	The overall economic level (e.g., regional GDP) in one region.
	Business Climate (C_23_)	The ability to attract enterprises, construction and investment.
	External communications (C_24_)	The regional economic openness and external links (e.g., external trade).
**Society** (**D_3_**)	Educational Level (C_31_)	The ability of regional talent cultivation and technology research cooperation.
	Development Potential (C_32_)	The potential of sustainable regional development.
	Public and Policy Support (C_33_)	The public goods and policy guidance that support the regional development.

**Table 2:** The initial influence matrix D for the criteria.

Criteria	C_11_	C_12_	C_13_	C_21_	C_22_	C_23_	C_24_	C_31_	C_32_	C_33_
C_11_	0.000	2.400	2.300	2.400	1.800	2.800	2.000	3.000	1.600	2.600
C_12_	2.200	0.000	3.100	2.400	1.200	2.200	2.400	2.000	2.800	1.800
C_13_	2.400	2.400	0.000	3.000	1.200	1.800	2.200	2.200	3.400	2.200
C_21_	3.400	2.200	2.700	0.000	2.600	3.200	2.200	2.200	2.600	2.400
C_22_	2.200	2.000	2.100	2.200	0.000	2.800	2.000	1.600	2.000	2.800
C_23_	2.200	1.800	2.300	2.400	2.400	0.000	2.000	2.600	2.400	1.600
C_24_	2.000	1.800	1.900	2.400	2.000	2.400	0.000	1.800	1.400	1.600
C_31_	2.400	2.400	2.300	2.200	1.600	2.400	2.800	0.000	1.400	2.200
C_32_	3.000	1.600	2.700	2.800	2.200	2.400	2.400	2.400	0.000	2.200
C_33_	2.600	2.000	1.300	2.200	2.200	2.000	1.800	1.800	1.800	0.000

**Table 3:** The normalized matrix R for the criteria.

Criteria	C_11_	C_12_	C_13_	C_21_	C_22_	C_23_	C_24_	C_31_	C_32_	C_33_
C_11_	0.000	0.094	0.102	0.145	0.094	0.094	0.085	0.102	0.128	0.111
C_12_	0.102	0.000	0.102	0.094	0.085	0.077	0.077	0.102	0.068	0.085
C_13_	0.098	0.132	0.000	0.115	0.089	0.098	0.081	0.098	0.115	0.055
C_21_	0.102	0.102	0.128	0.000	0.094	0.102	0.102	0.094	0.119	0.094
C_22_	0.077	0.051	0.051	0.111	0.000	0.102	0.085	0.068	0.094	0.094
C_23_	0.119	0.094	0.077	0.136	0.119	0.000	0.102	0.102	0.102	0.085
C_24_	0.085	0.102	0.094	0.094	0.085	0.085	0.000	0.119	0.102	0.077
C_31_	0.128	0.085	0.094	0.094	0.068	0.111	0.077	0.000	0.102	0.077
C_32_	0.068	0.119	0.145	0.111	0.085	0.102	0.060	0.060	0.000	0.077
C_33_	0.111	0.077	0.094	0.102	0.119	0.068	0.068	0.094	0.094	0.000

**Table 4:** The total influence matrix *T_C_*, determined by the criteria.

Criteria	C_11_	C_12_	C_13_	C_21_	C_22_	C_23_	C_24_	C_31_	C_32_	C_33_
C_11_	0.597	0.667	0.696	0.795	0.651	0.655	0.583	0.656	0.736	0.614
C_12_	0.597	0.490	0.599	0.648	0.555	0.552	0.497	0.569	0.589	0.512
C_13_	0.643	0.658	0.560	0.723	0.606	0.619	0.544	0.613	0.680	0.531
C_21_	0.678	0.664	0.705	0.655	0.641	0.652	0.588	0.639	0.717	0.590
C_22_	0.540	0.506	0.523	0.625	0.446	0.541	0.476	0.507	0.575	0.491
C_23_	0.693	0.656	0.663	0.777	0.662	0.560	0.589	0.647	0.704	0.585
C_24_	0.610	0.610	0.621	0.679	0.580	0.586	0.448	0.609	0.645	0.529
C_31_	0.646	0.597	0.622	0.681	0.568	0.607	0.521	0.503	0.647	0.530
C_32_	0.587	0.618	0.656	0.684	0.574	0.592	0.499	0.551	0.544	0.521
C_33_	0.620	0.577	0.610	0.676	0.600	0.562	0.504	0.577	0.629	0.450

**Table 5:** The total influence matrix *T_D_* of the dimensions.

Dimensions	D_1_	D_2_	D_3_
D_1_	0.612	0.619	0.611
D_2_	0.622	0.594	0.603
D_3_	0.615	0.589	0.550

**Table 6:** The sum of influences given to and received by the dimensions.

Dimensions	*r_i_*	*c_j_*	(*r_i_* + *c_j_*)	(*r_i_* − *c_j_*)
D_1_	1.842	1.849	3.691	−0.007
D_2_	1.819	1.802	3.621	0.017
D_3_	1.754	1.764	3.518	−0.010

**Table 7:** The unweighted supermatrix *W_C_*.

Criteria	C_11_	C_12_	C_13_	C_21_	C_22_	C_23_	C_24_	C_31_	C_32_	C_33_
C_11_	0.597	0.597	0.643	0.678	0.540	0.693	0.610	0.646	0.587	0.620
C_12_	0.667	0.490	0.658	0.664	0.506	0.656	0.610	0.597	0.618	0.577
C_13_	0.696	0.599	0.560	0.705	0.523	0.663	0.621	0.622	0.656	0.610
C_21_	0.795	0.648	0.723	0.655	0.625	0.777	0.679	0.681	0.684	0.676
C_22_	0.651	0.555	0.606	0.641	0.446	0.662	0.580	0.568	0.574	0.600
C_23_	0.655	0.552	0.619	0.652	0.541	0.560	0.586	0.607	0.592	0.562
C_24_	0.583	0.497	0.544	0.588	0.476	0.589	0.448	0.521	0.499	0.504
C_31_	0.656	0.569	0.613	0.639	0.507	0.647	0.609	0.503	0.551	0.577
C_32_	0.736	0.589	0.680	0.717	0.575	0.704	0.645	0.647	0.544	0.629
C_33_	0.614	0.512	0.531	0.590	0.491	0.585	0.529	0.530	0.521	0.450

**Table 8:** The weighted normalized supermatrix $W_{C}^{*}$.

Criteria	C_11_	C_12_	C_13_	C_21_	C_22_	C_23_	C_24_	C_31_	C_32_	C_33_
C_11_	0.365	0.365	0.394	0.420	0.335	0.429	0.378	0.395	0.359	0.379
C_12_	0.408	0.300	0.403	0.411	0.313	0.406	0.378	0.365	0.378	0.353
C_13_	0.426	0.367	0.343	0.436	0.324	0.410	0.384	0.380	0.401	0.373
C_21_	0.495	0.404	0.450	0.389	0.371	0.462	0.403	0.411	0.413	0.408
C_22_	0.405	0.345	0.377	0.381	0.265	0.394	0.345	0.343	0.346	0.362
C_23_	0.408	0.343	0.385	0.387	0.321	0.333	0.348	0.366	0.357	0.339
C_24_	0.363	0.309	0.338	0.349	0.283	0.350	0.266	0.314	0.301	0.304
C_31_	0.403	0.350	0.377	0.377	0.299	0.381	0.359	0.276	0.303	0.317
C_32_	0.452	0.362	0.418	0.423	0.339	0.415	0.380	0.356	0.299	0.346
C_33_	0.378	0.315	0.327	0.348	0.289	0.345	0.311	0.291	0.286	0.247

**Table 9:** The stable matrix of DANP.

Criteria	C_11_	C_12_	C_13_	C_21_	C_22_	C_23_	C_24_	C_31_	C_32_	C_33_
C_11_	0.410	0.346	0.381	0.392	0.314	0.392	0.355	0.350	0.344	0.343
C_12_	0.410	0.346	0.381	0.392	0.314	0.392	0.355	0.350	0.344	0.343
C_13_	0.410	0.346	0.381	0.392	0.314	0.392	0.355	0.350	0.344	0.343
C_21_	0.410	0.346	0.381	0.392	0.314	0.392	0.355	0.350	0.344	0.343
C_22_	0.410	0.346	0.381	0.392	0.314	0.392	0.355	0.350	0.344	0.343
C_23_	0.410	0.346	0.381	0.392	0.314	0.392	0.355	0.350	0.344	0.343
C_24_	0.410	0.346	0.381	0.392	0.314	0.392	0.355	0.350	0.344	0.343
C_31_	0.410	0.346	0.381	0.392	0.314	0.392	0.355	0.350	0.344	0.343
C_32_	0.410	0.346	0.381	0.392	0.314	0.392	0.355	0.350	0.344	0.343
C_33_	0.410	0.346	0.381	0.392	0.314	0.392	0.355	0.350	0.344	0.343

**Table 10:** Results Sort and Summary.

Criteria	Results	Sort
Transportation Conditions (C_11_)	0.410	1
Resource Reserves (C_12_)	0.346	7
Ecological Protection (C_13_)	0.381	4
Industrial Structure (C_21_)	0.392	2
Economic Scale (C_22_)	0.314	10
Business Climate (C_23_)	0.392	3
External communications (C_24_)	0.355	5
Educational Level (C_31_)	0.350	6
Development Potential (C_32_)	0.344	8
Policy Support (C_33_)	0.343	9
